# Psychotic relapse in people with schizophrenia within 12 months of discharge from acute inpatient care: protocol for development and validation of a prediction model based on a retrospective cohort study in three psychiatric hospitals in Japan

**DOI:** 10.1186/s41512-022-00134-w

**Published:** 2022-11-03

**Authors:** Akira Sato, Norio Watanabe, Kazushi Maruo, Toshihiro Moriyama, Toshi A. Furukawa

**Affiliations:** 1 Department of Health Promotion and Human Behavior, Kyoto University Graduate School of Medicine/School of Public Health, Yoshida-konoecho, Sakyo-ku, Kyoto, 606-8501 Japan; 2 Department of Psychiatry, Graduate School of Medicine, Kyoto University, 54 Shogoin-kawahara-cho, Sakyo-ku, Kyoto, 606-8507 Japan; 3 Department of Biostatistics, Faculty of Medicine, University of Tsukuba, 1-1-1 Tennodai, Tsukuba, Ibaraki, 305-8575 Japan; 4 Isogaya Hospital, 35 Isogaya, Ichihara, 290-0204 Japan

**Keywords:** Schizophrenia spectrum disorder, Hospitalization, Prognosis, Individualized risk

## Abstract

**Background:**

Schizophrenia is a severe mental illness characterized by recurrent psychoses that typically waxes and wanes through its prodromal, acute, and chronic phases. A large amount of research on individual prognostic factors for relapse in people with schizophrenia has been published, and a few logistic models exist to predict psychotic prognosis for people in the prodromal phase or after the first episode of psychosis. However, research on prediction models for people with schizophrenia, including those in the chronic phase and after multiple recurrences, is scarce. We aim to develop and validate a prediction model for this population.

**Methods:**

This is a retrospective cohort study to be undertaken in Japan. We will include participants aged 18 years or above, diagnosed with schizophrenia or related disorders, and discharged between January 2014 and December 2018 from one of the acute inpatient care wards of three geographically distinct psychiatric hospitals. We will collect pre-specified nine predictors at the time of recruitment, follow up the participants for 12 months after discharge, and observe whether our primary outcome of a relapse occurs. Relapse will be considered to have occurred in one of the following circumstances: (1) hospitalization; (2) psychiatrist’s judgment that the person needs hospitalization; (3) increasing doses of antipsychotics; or (4) suicidal or homicidal ideation or behavior resulting from such ideation. We will develop a Cox regression model and avoid overfitting by penalizing coefficients using the elastic net. The model will be validated both internally and externally by bootstrapping and “leave-one-hospital-out” cross-validation, respectively. We will evaluate the model’s performance in terms of discrimination and calibration. Decision curve analysis will be presented to aid decision-making. We will present a web application to visualize the model for ease of use in daily practice.

**Discussion:**

This will be the first prediction modeling study of relapse after discharge among people with both first and multiple episodes of schizophrenia using routinely collected data.

**Trial registration:**

This study was registered in the UMIN-CTR (UMIN000043345) on February 20, 2021.

## Background

Schizophrenia is one of the severe mental illnesses characterized by psychosis, such as delusions and hallucinations [1]. The lifetime prevalence of schizophrenia is estimated to be around seven in 1000, and the illness commonly renders people psychotic in their late adolescence and young adulthood [2]. A first psychotic episode in their life typically starts from the prodromal phase where they experience brief or attenuated psychotic symptoms. If the phase does not subside spontaneously, it develops into the acute phase, and people experience positive symptoms of psychosis. Following initial clinical contact, they usually reach remission after receiving antipsychotic treatment along with psychosocial support. People with the illness often have more than one psychotic episode during their lifetime where some experience a recovery and others experience a chronic phase [3]. The WHO conducted a prospective cohort study that followed 1633 participants worldwide with either schizophrenia or related disorders for more than 15 years in the 1970s and 1990s [4]. Only about 38% of those with schizophrenia and 55% of those with related disorders reached a recovery phase for a period of more than 2 years at the time of follow-up. Another study followed up 474 people with psychotic disorders, including schizophrenia, for 2 years after their discharge [5]. Of 149 participants who followed up at 2 years, 92 had relapsed, and 79 of those who relapsed were hospitalized, with the mean time to relapse being eight months.

Several studies of individual prognostic factors for relapse in schizophrenia have been conducted, as have the systematic reviews covering the topic [6, 7]. When focusing on an individual patient in daily practice, however, we realize that such a list of separately reported relative risks does not sufficiently inform the patient, their psychiatrist, and other mental health professionals about the patient’s prognosis. We need a prediction model that considers all relevant predictors simultaneously and provides the patient’s personalized risk. Unfortunately, research in prediction modeling in psychiatry has been scarce. A recent systematic review of prediction models in Psychiatry revealed that only 89 articles were published before 2019 [8]. Of those, only seven were regarding schizophrenia and one article developed a model for psychotic relapse based on predictors that could be assessable only in research-oriented, academic centers [9]. A few articles used covariates that are easily obtained in routine practice; however, most of them were models for people either in the prodromal phase or after the first episode only [10, 11], excluding those with schizophrenia after multiple episodes or in the chronic phase experiencing a relapse of florid psychotic symptoms on top of their chronic course.

We, therefore, aim to develop and validate a clinical prediction model that would estimate the risk of relapse in people with schizophrenia and with other related disorders, including those with multiple episodes or in the chronic phase, at the time of discharge from their acute inpatient care in psychiatric hospitals. Our primary interest is in building a practical, easy-to-use model with routinely collected data for people with such illnesses regardless of where they are in their lifetime trajectory.

## Methods

We will adhere to the Transparent Reporting of a Multivariable Prediction Model for Individual Prognosis or Diagnosis (TRIPOD) checklist for developing and validating our prediction model [12].

### Study design and source of data

Our prediction model will use datasets retrospectively obtained from three psychiatric hospitals in Japan that are distinct from each other in their physical venues and level of care. Chiba Psychiatric Medical Center (CPMC), located 30 km to the east of Tokyo, is a publicly owned tertiary care psychiatric facility that primarily treats psychosis. Urawa Psychiatric Sanatorium Hospital (UPSH) and Isogaya Hospital (IH) are private secondary care psychiatric hospitals 33 km to the north and 66 km to the east of Tokyo, respectively. CPMC, IH, and UPSH are in cities with 1.3, 1.0, and 0.27 million populations, respectively, with the former two being in the same regional government entity and UPSH in another.

How people receive psychiatric services in Japan is threefold, which may or may not allow some people to be admitted to more than one of the three hospitals. Firstly, the Japanese healthcare system allows people to self-refer to psychiatrists at an outpatient clinic of, for example, these psychiatric hospitals. Secondly, people can contact a local mental health helpline to find a hospital for psychiatric urgency during nights and weekends when outpatient clinics are closed. Thirdly, people can call the police or an ambulance in a mental health emergency. Since each regional government operates the latter two services, people using the services can be admitted to hospitals within each region. Therefore, we cannot rule out the possibility of some patients being admitted in both CPMC and IH in the same region, while there is less chance for UPSH to experience such patient overlap. We will try to exclude such possibilities by checking the potential overlaps among our cohorts.

UPSH and IH constitute several wards for mental illnesses in both acute and chronic conditions, whereas CPMC has a ward for acute care only. We will only collect data from acute inpatient care wards in these participating hospitals with about 200 beds in total. In Japan, people experiencing an acute exacerbation of schizophrenia or related disorders are typically treated in these wards, with the average length of stay being 56.7 days from 2011 onwards [13, 14]. On the contrary, the average length of stay for all psychiatric beds was very long at 298 days in 2011 [13], suggesting that a high number of non-acute care beds in Japan provided care for long-stay patients [15], and these beds may not be regarded as mental health beds in other countries [13]. Organization for Economic Cooperation and Development (OECD) has reported that “there is reason to believe that the patients in long-stay beds could be effectively cared for in their homes or in the community” [16]. We will, therefore, not collect data from non-acute care wards, which are unlikely to provide treatment for people with acutely relapsing psychotic disorders.

We will use the same dataset for both the development and validation of our prediction model.

### Study population

People with schizophrenia and related disorders who were discharged from an acute inpatient ward in the three psychiatric hospitals between 1 January 2014 and 31 December 2018 will be recruited in a consecutive manner.

We chose this 5-year period to avoid major concurrent events of the disastrous earthquake and novel coronavirus pandemic in 2011 and 2019, respectively. The number of psychiatric hospitalizations increased among people with schizophrenia in the affected areas of eastern Japan after a severe earthquake hit the region on 11 March 2011 [17]. A psychiatric hospital in Tokyo, about 350 km away from the epicenter, observed that the number of involuntary admissions among people with schizophrenia increased after the earthquake, whereas the number among people with other mental illnesses did not increase [18]. The novel coronavirus disease pandemic, which started in December 2019, might also affect the relapse of severe mental illnesses [19, 20].

### Eligibility criteria

Our inclusion criteria for this study are as follows: age 18 years or older; a diagnosis of schizophrenia and related disorders, including schizophrenia, schizotypal disorder, persistent delusional disorders, acute and transient psychotic disorders (ATPD), induced delusional disorder, schizoaffective disorders, other nonorganic psychotic disorders, and unspecified nonorganic psychosis; inpatient care provided primarily to treat psychosis; and discharge from an acute inpatient care ward. We will use the international classification of diseases 10th revision (ICD-10) for the diagnosis [1]. If a participant has several hospitalization episodes during the study period, we will randomly choose one.

We will exclude people with substance/medication-induced psychotic disorder; psychotic disorder due to another medical condition; with an apparent diagnosis of schizophrenia or related disorders without fully meeting the criteria; currently admitted for reasons other than psychosis; discharged from a non-acute ward; with an unclear diagnosis; transferred to another psychiatric/medical facility; and with an immediate plan to return home overseas after discharge. We will record reasons for exclusion for all hospitalization episodes along with their age and sex.

The accuracy of our eligibility criteria will be assessed by two investigators independently deciding which potential participants to be included or excluded. We will do this for 30 consecutive potential participants and present a kappa statistic for the inter-rater agreement.

### Study outcome

Our primary outcome will be psychotic relapse within 12 months after discharge from the hospitals. We will follow up the participants after discharge by reviewing their medical records to observe whether they have had a relapse. We will define relapse as an occurrence of any one of the following: (1) hospitalization; (2) psychiatrist’s judgment that the patient requires hospitalization; (3) increasing doses of antipsychotics; or (4) suicidal or homicidal ideation or violent behavior resulting in injury to self or another person. All events should occur because of psychotic exacerbation. Additional details of each component of our definition of relapse are described below. For participants who are lost to follow-up, we will endeavor to identify and record the reason.

We will define hospitalization as a patient being admitted to a psychiatric hospital based on a physician’s decision that the patient needs inpatient care to treat psychotic exacerbation. While such inpatient care will usually be provided by one of the participating hospitals, we will attempt to follow up any hospitalization at another hospital, by finding, within our medical records, any reports about such hospitalization by, for example, mental health professionals or public health officials outside the participating hospitals. Instances where a physician examining a patient’s mental status decides that the patient needs to be hospitalized to treat their psychosis but the patient disagrees and, hence, is not admitted will be included as a psychotic relapse because the patient is in a state of psychotic exacerbation that should not be overlooked. We will define increasing doses of antipsychotics as (1) any increase in the current antipsychotic dose for more than seven consecutive days, (2) the addition of a new antipsychotic to the current one for more than seven consecutive days, (3) switching the current antipsychotic with another and if the chlorpromazine equivalent dose of the new antipsychotic is increased for more than seven consecutive days, or (4) addition of an as-needed prescription of an antipsychotic that is used for more than either five out of seven consecutive days or 10 out of 14 consecutive days. Increasing the dose of antipsychotic medications should be based on the intention to treat psychotic worsening, and any changes in the antipsychotic dosage and its reason will be reviewed in the medical records. Suicidal or homicidal ideation and violence resulting in injury to self and/or others will be defined in terms of an occurrence of more than or equal to two such events within a month, or a single event if the police and/or ambulance staff are involved. If any of these events happen under the patient’s decreased level of reality testing within seven days prior to the events and stated as such in the medical records, we will include them.

Hospitalization, per se, is not our primary outcome because it does not necessarily cover all psychotic relapses. We, therefore, decided to include several aspects of psychotic relapse, such as non-hospitalized relapse or relapse that is successfully treated by increasing the antipsychotic dose before it leads to readmission. We believe that the inclusion of these relapses will capture psychotic exacerbation more adequately than hospitalization alone.

### Selection of candidate predictors

We pre-specified candidate predictors based on the literature and expert opinions. First, we systematically searched previous literature, screened 3490 abstracts, and counted the number of each predictor that appeared in 189 articles identified for data extraction (see Appendix) [21]. Focusing on routinely collected predictors that were identified as such in more than ten studies, we identified nine potential predictors; age, sex, previous hospitalizations, relapse or hospitalizations in the preceding year, the current length of stay, any substance use disorders, alcohol use disorder, use of long-acting injections, and psychosocial interventions. We combined all substance use disorders, as substances other than alcohol are seldom abused in Japan. Based on expert opinion, we decided to have another predictor, the receipt of benefits, and agreed that predictors other than those listed above were unnecessary. We will, therefore, include nine candidate predictors for our prediction model (see Table 1).

**Table 1:** Candidate predictor variables for psychotic relapse

Predictor	Definition	Variable type	Units/categories
Age	Patient’s age at the time of discharge	Continuous	Years
Sex	Participant’s sex as recorded in the medical records	Binary	Male, female
Previous hospitalizations	The total number of psychiatric hospitalizations in the past, irrespective of their duration, reasons, and whether they were involuntary	Continuous	Count
Hospitalizations in the previous year	Presence of any hospitalizations for treating psychosis in the past 12 months before the start of the current admission, excluding hospitalizations for reasons other than psychosis	Binary	Yes, no
Current length of stay	The total number of days spent hospitalized for the current episode of psychosis	Continuous	Days
Substance use disorders	Presence of any current substance use disorder, including alcohol, as clearly mentioned as such in the medical records	Binary	Yes, no
Use of LAI	Presence of any use of antipsychotic LAI at the time of discharge	Binary	Yes, no
Psychosocial interventions	The total number of any psychosocial sessions a patient received regardless of the duration per intervention, including, for example, psychoeducational, social skill training, and occupational approaches, and excluding those provided to the participant's family members	Continuous	Count
Receipt of benefits	Presence of the receipt of benefits from a local government, as shown in the medical records	Binary	Yes, no

*LAI* long-acting injection

We will code these variables from the viewpoint of minimizing the loss of information by unnecessary categorization. Age will be recorded at the discharge date as continuous variable. Sex will be recorded as a binary variable. Previous hospitalizations will be recorded as the total number of psychiatric hospitalizations in the past. We will include any psychiatric hospitalizations regardless of the duration, whether they were compulsory, or the reasons for inpatient care because it is not feasible to discriminate these events in our retrospective study. Relapse or hospitalization in the previous year will be treated as a binary variable. Our study will only include hospitalization and refer to this variable as such hereafter because measuring non-hospitalized relapse retrospectively is not possible in our study design. We will examine whether a patient has been hospitalized to treat psychotic exacerbations in the past 12 months before the start of current hospitalization and attempt to exclude any hospitalizations for reasons other than psychosis, such as social issues not directly related to exacerbation of positive symptoms. The length of stay of the index hospitalization will be recorded as the number of days spent hospitalized. Any substance use disorder will be treated as a binary variable. Use of a long-acting injection (LAI) at the time of discharge will be recorded as a binary variable. Psychosocial interventions will be recorded as the number of times a patient received such interventions regardless of the duration per intervention. Psychosocial interventions will include, but not limited to, psychoeducational, social skills training, and occupational therapeutic approaches. We will exclude any interventions provided to their family members as they are not recorded in our data sources. The receipt of benefits from a local government will be recorded as a binary variable.

We will present the numbers and proportions for each binary variable and all missing data. For continuous variables, we will report the mean and standard deviation if the data are distributed normally and the median and interquartile range if it has a skewed distribution.

### Data extraction

We will first extract candidate predictors and auxiliary variables for the included participants by reviewing their inpatient records. For most of our data sources, inpatient and outpatient medical records are separately stored at physically different places. When extracting predictors from inpatient records, we will be blinded to an outcome occurrence that is documented in outpatient records. We will report the proportion of unblinded outcome data wherever possible.

We will extract an outcome by following up on eligible participants in their outpatient records for 12 months after discharge. When recording the outcome data, we will attempt not to review predictors by creating two separate datasets with different data entry sheets. We will present the proportion of unblinded data regarding predictors by recording the number of times we happen to know the status of any predictors while collecting the outcome.

To assess the reliability of data extraction, two investigators will independently collect data for 30 consecutively included participants for predictors and outcomes. We will present percentage agreements and kappa statistic for binary variables and an intraclass correlation coefficient (ICC) for continuous variables.

### Data cleaning

We will create box plots with the median and interquartile range (IQR) for the continuous variables. We will then identify any outliers that lie either below the one or above the 99 percentiles. Should we have such outliers, we will first check if errors in entering data occurred by reviewing a participant’s record and correcting them accordingly. For outliers deemed plausible, we will transform them by “winsorizing” that shifts those outliers in very low or very high values to the 1 or 99 percentiles, respectively.

If we have predictors with a very narrow distribution (e.g., ≥99 % of participants being of one sex), we will discuss whether to eliminate or include them and report these and their reasons [22, 23]. Should we have predictors with a very skewed distribution, we will discuss whether to omit them or include them by taking a log-transformation or whether to exclude participants with such predictors and present them as such [22].

### Sample size calculation

Using the criteria suggested by Riley et al .[24], we calculated the minimum sample size needed to avoid overfitting the predictor effects in our model development. We required a Cox-Snell R^2^ to estimate the sample size, which can be derived from the C statistic in previous studies. While no previous studies dealt with the same or very similar population for the same outcome as in our study, we managed to have a Cox-Snell R^2^ of 0.10 after employing a C statistic of 0.66 obtained from a study that validated a model for an outcome of 2-year risk of relapse in people with first-episode psychosis [11]. We also obtained a rate of relapse (37.7%) and a mean follow-up time (1.59 years) for people with first-episode psychosis in another study [25]. With the Cox-Snell *R*^2^, rate, mean follow-up time, and nine parameters in our list of variables, we needed a minimum of 754 participants for our prediction model, corresponding to an event per parameter (EPP) of 50.

The numbers of patients admitted to the acute care wards per year between 2014 and 2018, on average, were 367, 160, and 368 for CPMC, IH, and UPSH, respectively. Our preliminary search revealed that about 32%, 19%, and 15% of those admitted to CPMC, IH, and UPSH were potentially eligible for our study; the approximate sample size would be about 1015. This sample size will allow us to include some additional parameters, for example, for interactions or nonlinearity described below.

### Missing data

We decided that predictors with more than 50 % missing data be discarded from our prediction model. We will plan a sensitivity analysis comparing a model excluding the omitted variables with a model including them. We will impute missing values using multiple imputation with chained equations for the remaining predictors in our dataset [26]. Our imputation (regression) model will include auxiliary variables and the outcome along with predictors. Auxiliary variables will include calendar year, physical venue of the hospitals, types of admission (i.e., voluntary or compulsory), current smoking status, and body mass index (BMI). We will create 20 imputed datasets, analyze each of them separately, and combine these 20 analyses to obtain the final estimates.

### Model development

We will apply a Cox regression model to predict a composite outcome for psychotic relapse. We will treat both participants who drop out before the end of the study and who have no relapse at the 12-month follow-up as censored. We will assess the additivity and linearity assumption by performing overall tests for all interactions and nonlinearity. We will explore specific interaction and nonlinear terms to be included in our model if the test’s *p*-value is less than 0.05. We may consider using a smart coding as described by Steyerberg for interaction terms [27]. We will examine the nonlinearity of the continuous variables using transformations such as the squared, log, and restricted cubic splines. We will assess this flexible model in our sensitivity analysis. To avoid overfitting, we will employ the elastic net for a penalized estimation of regression coefficients [28, 29]. The elastic net allows both selection and penalization of main effects by introducing two tuning parameters. It also considers correlations between predictors. Optimal values for the two parameters will be obtained through a tenfold cross-validation.

### Model performance

For our model performance, we will evaluate overall accuracy, discrimination, and calibration. For overall accuracy, that is, to what extent the prediction model can explain the amount of variability in outcomes, we will calculate *R*^2^ and the Brier score at fixed time points [30]. For discrimination, namely, the ability of a model to discriminate participants with the outcome from those without the outcome, we will calculate the C statistic and demonstrate the separation between prognostic groups by calculating the D statistic or illustrating graphically using a grouped Kaplan–Meier plot [30–33]. We will evaluate calibration, the agreement between the observed and predicted outcomes, by calculating a calibration slope and a calibration plot [30, 34]. Wherever possible, we will report optimism-corrected performance measures derived from subtracting optimism from apparent performance in the original dataset. We will calculate optimism using bootstrapping procedure as described by Steyerberg [35]. We will perform decision curve analysis (DCA) to help decision-making by setting the cut-off in the context of users among predicted probabilities and compare clinical usefulness among several models in sensitivity analyses [36].

### Model validation

We will examine both internal and external validity [37]. Bootstrap validation with 500 repetitions will be performed to assess the model’s reproducibility. Geographical transportability will be inspected by “leave-one-hospital-out” cross-validation. In this internal-external validation, a dataset from one hospital out of the three will be left out to test a model’s performance, and a dataset of the remaining two hospitals will be used to construct the model. This process will be repeated for each of the two hospitals similarly. We will endeavor to identify the amount of heterogeneity between these hospitals.

### Sensitivity analyses

We will develop a model with a single outcome of hospitalization as our secondary outcome by narrowing our definition of relapse. We will perform other sensitivity analyses for our primary outcome to observe whether our model’s performance changes. We will develop three prediction models for people with schizophrenia, excluding people with other psychotic disorders, people with first-episode schizophrenia, and people aged between 18 and 65, excluding older adults over 65 years. We will develop another model using datasets including variables discarded because of a high proportion of missingness. We will assess how its performance changes by comparing it with the original model without such variables. We will also compare a flexible model with interactions and nonlinearities with a simple model without them if we were to develop such a complex model.

### Statistical software

We will use R version 4.0.3 or above for our analysis [38]. We will plan to create a web-based application using a “Shiny” package of R. People with schizophrenia, their psychiatrists and other mental health professionals can use this “web app” to understand with ease the personalized risk of psychotic relapse without juggling variables and their coefficients.

## Discussion

We have described the protocol for a prediction model development study for 12-month risk of psychotic relapse in people with schizophrenia. To our knowledge, this is the first prediction modeling study focusing on people with both first and multiple episodes of schizophrenia and other psychoses, reflecting a real setting in daily practice. We selected predictors among routinely collected data because intended users of our model can easily obtain such “inputs” from their everyday practice. Regarding geographical validation, the strength of our study lies in the three data sources that are quite distinct from each other. Our prediction model will possibly provide people with schizophrenia and their mental health professionals with an additional resource of personalized risk that helps them decide what extra care they can choose to prevent relapse should our model show good performance.

Our study is not without limitations. First, due to its retrospective nature, we will not be able to collect some variables. For example, the family’s expressed emotion directed toward the patient is a significant predictor of relapse despite not being recorded in our data sources [39]. Second, our study may underestimate psychotic relapse. Some patients can be readmitted to another psychiatric facility other than the participating hospitals, and we may not know this occurrence despite our extended efforts as described above. The doctors’ judgment that the patient needs hospitalization and occurrence of violent behaviors may also not be explicitly recorded. Finally, our primary outcome is unlikely to take into full account the quality of life during the non-relapse period. We will also not be able to capture negative symptoms, such as avolition and anhedonia, that often lower the patients’ quality of life and interfere with their activities of daily living.

Should our prediction model have poor performance or sensitivity analyses suggest further improvement, we will plan to update the model by, for example, prospectively collecting important variables or adding other data sources.

## Data Availability

The datasets to be used during the current study are not publicly available because individual privacy could be compromised.

## Appendix

Search terms used to screen published articles for potential predictors

We searched Ovid Medline on 3 September 2020 with search terms for each concept of schizophrenia, relapse, and prediction. Search terms for each concept, including synonyms, are combined with a Boolean operator “OR”, which are then combined with a Boolean operator “AND” as described below. For the concept of prediction, we used a search filter recommended by Ingui et al. [41]. We identified 3490 articles for screening and included 189 articles for data extraction.

1. ((Validat* OR Predict*.ti. OR Rule*) OR (Predict* AND (Outcome* OR Risk* OR Model*)) OR ((History OR Variable* OR Criteria OR Scor* OR Characteristic* OR Finding* OR Factor*) AND (Predict* OR Model* OR Decision* OR Identif* OR Prognos*)) OR (Decision* AND (Model* OR Clinical* OR Logistic Models/)) OR (Prognostic AND (History OR Variable* OR Criteria OR Scor* OR Characteristic* OR Finding* OR Factor* OR Model*)))
2. (schizophrenia* OR schizophrenic* OR schizophreniform OR paranoid* OR delusional OR catatoni* OR hebephreni* OR psychos#s OR psychotic* OR schizoaffective*)
3. (relapse* or hospitali#ation* or recurren* or readmission*)
4. 1 and 2 and 3

## Abbreviations

ATPD: Acute and transient psychotic disorder
BMI: Body mass index
CPMC: Chiba Psychiatric Medical Center
DCA: Decision curve analysis
EPP: Event per parameter
ICC: Intraclass correlation coefficient
ICD-10: International Classification of Diseases 10th revision
IH: Isogaya Hospital
IQR: Interquartile range
LAI: Long-term injection
OECD: Organisation for Economic Cooperation and Development
TRIOPOD: Transparent Reporting of a Multivariable Prediction Model for Individual Prognosis or Diagnosis
UMIN-CTR: University Hospital Medical Information Network Clinical Trial Registry
UPSH: Urawa Psychiatric Sanatorium Hospital
WHO: World Health Organization
